# Unveiling the Fecal Microbiota in Two Captive Mexican Wolf *(Canis lupus baileyi)* Populations Receiving Different Type of Diets

**DOI:** 10.3390/biology10070637

**Published:** 2021-07-09

**Authors:** Sergio I. Barraza-Guerrero, César A. Meza-Herrera, Cristina García-De la Peña, Verónica Ávila-Rodríguez, Felipe Vaca-Paniagua, Clara E. Díaz-Velásquez, Irene Pacheco-Torres, Mónica A. Valdez-Solana, Quetzaly K. Siller-Rodríguez, Luis M. Valenzuela-Núñez, Juan C. Herrera-Salazar

**Affiliations:** 1 Facultad de Ciencias Biológicas, Universidad Juárez del Estado de Durango, Gómez Palacio 35010, Mexico; sergiokun.barraza@gmail.com (S.I.B.-G.); vavilar@gmail.com (V.Á.-R.); qksr@hotmail.com (Q.K.S.-R.); luisvn70@hotmail.com (L.M.V.-N.); hsjc20@ujed.mx (J.C.H.-S.); 2 Unidad Regional Universitaria de Zonas Áridas, Universidad Autónoma Chapingo, Bermejillo 35230, Mexico; cmeza2020@hotmail.com; 3 Laboratorio Nacional en Salud: Diagnóstico Molecular y Efecto Ambiental en Enfermedades Crónico-Degenerativas, Facultad de Estudios Superiores Iztacala, Universidad Nacional Autónoma de México, Tlalnepantla 54090, Mexico; felipe.vaca@gmail.com (F.V.-P.); cdiazvelasquez@aol.com (C.E.D.-V.); 4 Instituto Nacional de Cancerología, Ciudad de México 14080, Mexico; 5 Unidad de Biomedicina, Facultad de Estudios Superiores Iztacala, Universidad Nacional Autónoma de México, Tlalnepantla 54090, Mexico; 6 Programa de Posgrado en Recursos Genéticos y Productividad-Ganadería, Colegio de Postgraduados, Campus Montecillo, Km. 36.5 Carretera México-Texcoco, Montecillo 56230, Mexico; irenepacheco.t@gmail.com; 7 Facultad de Ciencias Químicas, Universidad Juárez del Estado de Durango, Gómez Palacio 35010, Mexico; valdezandyval@gmail.com

**Keywords:** microbiota, wolf, Michilia, Ocotal, diet, conservation

## Abstract

**Simple Summary:**

The Mexican wolf (*Canis lupus baileyi*) is an endangered canine. Both Mexico and the United States are currently collaborating to reproduce and reintroduce individuals to their original habitats. However, keeping these wolves in captivity represents a great commitment to meet their basic needs. Diet is a determining factor that is closely related to health and reproductive fitness. The type of diet that is fed to canines in captivity must provide the required nutrients for their development and welfare. The study of the fecal microbiota is a non-invasive way to establish the abundance and diversity of bacterial communities to determine if they are in a healthy condition. We analyzed data from two captive populations of Mexican wolves (i.e., northern and central Mexico) receiving different type of diets (Michilia population: mainly kibble vs. Ocotal population: mainly raw meat). The operational taxonomic units (OTUs) in Michilia resulted in 204 genera and 316 species, while in Ocotal there were 232 genera and 379 species. In the Michilia, dominance of bacteria that degrade carbohydrates was observed (related to kibble diet). In contrast, the Ocotal microbiota was dominated by protein-degrading bacteria (related to raw meat diet). The main outcomes generated in this study should help to enhance the welfare of the captive Mexican wolves to increase its numbers.

**Abstract:**

The Mexican wolf (*Canis lupus baileyi*) was once distributed in southern United States and northern Mexico. It is an endangered subspecies detached from the gray wolf, and likely exemplifies one of the original migration waves of *C. lupus* into the new world. This is a canine whose individuals survive in specialized facilities, zoos, and museums as part of captive-breeding programs. In order to contribute to the improvement of the management of this species and favor its long-term conservation in Mexico, we aimed to evaluate the diversity and abundance of the fecal bacterial microbiota in two populations exposed to different types of diet: (1) Michilia (23° N, 104° W); kibble daily and raw meat sporadically, and (2) Ocotal (19° N, 99° W); raw meat daily and live animals periodically. Next generation sequencing (V3-V4 16S rRNA gene) by Illumina was implemented. The operational taxonomic units (OTUs) in Michilia resulted in 9 phyla, 19 classes, 34 orders, 61 families, 204 genera, and 316 species, while in Ocotal there were 12 phyla, 24 classes, 37 orders, 69 families, 232 genera, and 379 species. Higher estimated Chao1 richness, Shannon diversity, and core microbiota were observed in Ocotal. Differences (*p* < 0.05) between populations occurred according to the Bray–Curtis beta diversity index. In the Michilia, dominance of bacteria that degrade carbohydrates (Firmicutes, Lachnospiraceae, *Blautia*, *Clostrodium*, *Eisenbergiella*, *Romboutsia*, and *Ruminococcus*) was observed; they are abundant in kibble diets. In contrast, the Ocotal microbiota was dominated by protein-degrading bacteria (Fusobacteria, Fusobacteriaceae, and *Fusobacteria*), indicating a possible positive relation with a raw meat diet. The information generated in this study is fundamental to support the implementation of better management plans in the two populations considered here, as well as in different facilities of southern United States and Mexico, where this subspecies is kept in captivity for conservation purposes.

## 1. Introduction

The intestinal bacterial microbiota plays an important role in health and welfare of vertebrates. These bacteria, directly and indirectly, affect the physiology, immune system, and nutrition of the host through different mechanisms of biological coexistence such as mutualism, commensalism, and pathogenicity [1,2,3]. Some benefits provided by the intestinal microbiota are the following: it is a first defense barrier against pathogenic bacteria, promotes intestinal development, enhances fermentative processes to make substrates more easily digested, and produces vitamins for the host [4,5]. To recover threatened species, one of the most used strategies is bringing animals into captivity and intensively managing its populations [6]. However, captive management of wild populations implies to have not only a great knowledge of the biology and ecology of the species, but increased economic resources to try to recreate their free-life original inhabit conditions. In this respect, providing an adequate diet to a large canine species is one of the most challenging aspects for conservation organizations, as most of the time it is far from the nutritional composition they obtain in their natural environment [7]. In several canine species (red wolf, gray wolf, and dogs), diet changes have shown microbiological diversity and abundance alterations due to the different type of available substrates to digest [8,9,10,11]. As the imbalance of the intestinal bacterial communities may, in some cases, be associated with severe negative health consequences for the host [12], conservation efforts for threatened species kept in captivity have included the analysis of the host’s fecal microbiota to monitor their health and promote their survival [6,7,12].

The Mexican wolf (*Canis lupus baileyi*) is considered a subspecies separated from the gray wolf, and likely represents one of the earliest waves of migration of *C. lupus* into the new world [13]. It is an endemic species of Mexico [14], divergent form all other North American wolves’ populations, suggesting that its presence in the Americas arises from a different colonization history regarding the remaining North American gray wolves [15]. Due to massive hunting by cattlemen in northern Mexico and southern USA, carried out at the beginning of the 20th century, this canine is currently considered extinct in free life [16,17]. A binational recovery program has been developed to reestablish Mexican wolves in Mexico and the USA [18]; this species had a great ecological importance because as a predator, it shaped the structure of biological communities, preserving diverse populations of plants and animals [19]. Currently, it is an emblematic species used as insignia for the conservation of biodiversity in Mexico [16]. Today, there are several specialized facilities, zoos, and museums that participate in the raising of Mexican wolves as part of the binational recovery program. In Mexico, there is a captivity facility located in the La Michilia Biosphere Reserve in the state of Durango, and another in the Ocotal State Park in the State of Mexico. The diet provided to the wolves in each of these facilities is different: (Michilia, Durango: kibble daily and raw meat sporadically, vs. Ocotal, State of Mexico: raw meat daily and live animals periodically). As the type of diet is an important factor in the fecal microbiota of canines, we quantified the diversity and abundance of the fecal bacterial microbiota (16S rRNA gene amplicon sequencing) of these two Mexican wolves’ populations. We hypothesized a significant difference in the fecal bacterial microbiota between Michilia and Ocotal. The information generated in this study should contribute to improve the management policies and practices of captivity programs to preserve the Mexican wolf, benefiting its welfare and fitness, while favoring its long-term conservation.

## 2. Materials and Methods

### 2.1. General

All the methods and activities of this study were in strict accordance with accepted guidelines for ethical use, care, and welfare of animals in research at the international level [20]. The federal approval reference number is SEMARNAT SGPA/DGVS/05117/17.

### 2.2. Location, Experimental Sites, Environmental Conditions and Animal Diets

*Michilia population.* There is a biological station within the La Michilia Biosphere Reserve (23°15′ and 23°35′ N, 104° and 104°20′ W), located in the municipality of Suchil, southeast Durango, Mexico. The average altitude is 2480 m above sea level (m.a.s.l.) and the precipitation fluctuates between 600 and 860 mm. There are two thermal zones, a temperate area located in the northern part, and on the slopes of hills (2700 m.a.s.l.) with an average annual temperature between 12 and 18 °C. The other is a semi-cold area at altitudes greater than 2700 m with an average annual temperature below 12 °C [21]. The vegetation profiles fluctuate along with the altitude. The dominant vegetation is composed of pine-oak forest (*Pinus* spp. and *Quercus* spp.) and other tree species such as *Pseudotsuga* spp., *Cupressus* spp., and *Juniperus* spp. to a lesser extent [21]. This biological station involves three enclosures (≈2000 m^2^ each), where Mexican wolves are held. The diet offered for wolves in this location consists of commercial dry dog food (kibble; 16% protein, 7% fat, 12% water, 8% ash, 57% carbohydrates) on a daily basis, and fresh meat every 10–15 days. These wolves are totally isolated from the closest community, and have no contact with human beings, with the exception of the keeper who feeds them.

*Ocotal population.* The Ocotal State Park (19°48 and 19°47′ N, 99°45′ and 99°45′ W) is located in the municipality of Timilpan, State of Mexico, Mexico, within the Transversal Volcanic system. The area is located at an altitude of 2750 m.a.s.l., and occupies an area of 122.14 ha. In this region the climate is temperate, sub-humid, with precipitation greater than 550 mm [22]. In this park, the pine-ocote (*Pinus oocarpa*) predominates and there are also areas of oaks and grasslands [22]. In this location, the wolves are kept in a pack within an area close to 3500 m^2^; the wolves receive a diet consisted of fresh meat, and once a week of live rabbits or guinea pigs. These wolves are in a special area restricted to the public, where they only have contact with several keepers and veterinarians who care for them.

### 2.3. Sampling According to Gender, Age, and Type of Animal Grouping

In both geographical sites, animals were monitored at a distance of 10 m during the morning (800 to 1000 h), awaiting the fecal evacuation. Stool samples were taken no more than 30 min after deposition. Stools that showed firm consistency were “opened” to sample the interior; for the watery feces, a sample was taken from the surface to avoid soil contamination. For each stool, approximately 0.3 g was collected in a BashingBead™ Zymo Research™ tube, adding 750 µL of lysing solution Zymo Research™ stabilizer. Each tube was processed on a cell disruptor (TerraLyzer™ Zymo Research Corp., Irvine, CA, USA) for 30 s and transported to the lab in an ice box in the next four hours. The collected stool samples information regarding sex, age, and type of grouping are shown in Table 1.

### 2.4. DNA Extraction and Visualization

The QIAGEN DNeasy Blood and Tissue kit was used to extract the fecal DNA using a UV laminar flow hood with all sterility protocols. The DNA extraction products were run on 1.2% agarose gels at 80 V for 45 min in a Bio-Rad electrophoresis chamber to visualize the presence of DNA. Visualization was carried out on a GelMax^TM^ photodocumenter (UVP^®^, Upland, CA, USA). The concentration and quality of DNA obtained from the samples was measured on a Qubit^®^ 3.0. (Invitrogen, Carlsbad, CA, USA).

### 2.5. 16S rRNA Gene Amplicon Sequencing

Amplification of the V3-V4 region of the 16S rRNA gene was made using the following primers [23]: S-D-Bact-0341-b-S-17 5′-CCTACGGGNGGCWGCAG-3′ and S-D-Bact-0785-a-A-21 5′-GACTACHVGGGTATCTAATCC-3′. Subsequently, the Illumina PCR protocol, Illumina [24] was implemented using 12.5 µL of MyTaq^TM^ Ready Mix 1X (Bioline^®^, London, UK), 1 µL of each primer (10 nM), 5 µL of DNA (25 ng total) and 5.5 µL of ultrapure H_2_O; the following cycle was used: 95 °C for 3 min; 25 cycles of 95 °C for 30 s, 55 °C for 30 s, 72 °C for 30 s; and 72 °C for 5 min in a Labnet Multigene^TM^ Gradient PCR (Labnet International, Inc. Global, Edison, NJ, USA) thermal cycler. Amplicons were purified with 0.8% Agentcourt^®^ AMPure^®^ XP beads (Beckman Coulter Inc., Brea, CA, USA). Then, the amplicons were labeled using Nextera XT Index Kit^TM^ (Illumina, Inc., San Diego, CA, USA) for the creation of the libraries, following the Illumina protocol [25], using 25 µL of MyTaq^TM^ Ready Mix 1X (Bioline^®^), 5 µL of each primer (N7xx and S5xx), 5 uL of DNA, and 10 µL of ultrapure H_2_O; the following cycle was used: 95 °C for 3 min; 10 cycles of 95 °C for 30 s, 55 °C for 30 s, 72 °C for 30 s; and 72 °C for 5 min. Finally, quantification, normalization (equimolarity), library pooling, and next-generation massive sequencing (MiSeq, Illumina, San Diego, CA, USA) of 2 × 250 paired end reads) were performed following the Illumina 16S protocol [24].

### 2.6. Bio-Informatic Analyses

Sequence analysis was performed using Quantitative Insights into Microbial Ecology (QIIME) [26]. The assembly was made using PEAR [27] with Q30. Chimeras were removed with USEARCH [28]. Operational taxonomic units (OTUs) were selected using UCLUST [28] at 97% similarity; taxonomy was assigned using EzBioCloud database [29]. Random rarefaction was carried out at a depth of 13,050 sequences. From here, the Chao1 estimated richness index and the Shannon alpha diversity index were calculated. Non-parametric *t*-tests (false discovery rate correction) were applied to test differences (*p* < 0.05) between populations for each index. The Bray–Curtis beta diversity [30] was calculated; PERMANOVA was applied to test significant differences (*p* < 0.05) of the fecal microbiota between populations, and it was visualized using principal coordinate analysis (PCoA) in Emperor [31]. The relative bacterial abundance was obtained at all taxonomic levels. The most abundant phyla and families were represented in stacked bar graphs using R, and the genera were visualized in a heatmap using Morpheus (https://software.broadinstitute.org/GENE-E/; accessed 15 January 2021). To establish the bacterial taxa at phylum, family and genus levels that contributed the most to the differentiation of the fecal microbiota between both populations, a percentage similarity analysis SIMPER [32] was developed using the Bray–Curtis matrix in PAST 4.0. For those taxa whose contribution was greater than 0.2%, a Mann–Whitney U test (*p* < 0.05) was applied to test for significant differences between populations. We also determined the core microbiota (those taxa that are found in all individuals) for each population. Finally, a LEfSe (linear discriminant analysis effect size) analysis was performed to statistically and biologically determine the key biomarkers which contribute the most to the differences between populations. The clades selected were those less than 0.05 in the alpha value of the Kruskal–Wallis factorial test > 4.0 in the logarithmic LDA score [33]. This analysis was made on the website http://huttenhower.sph.harvard.edu/lefse/ (accessed 25 January 2021).

## 3. Results

The mean of assembled sequences for the Michilia wolves was 38,402, and for Ocotal was 56,663. The mean of quality bacterial sequences in the Michilia samples was 26,467, and 37,043 for the Ocotal samples. The mean OTUs were 1475 and 3449, respectively (Table 2). The OTUs in the Michilia population resulted in 9 phyla, 19 classes, 34 orders, 61 families, 204 genera, and 316 species, while in Ocotal there were observed 12 phyla, 24 classes, 37 orders, 69 families, 232 genera, and 379 species.

**Figure 1:**
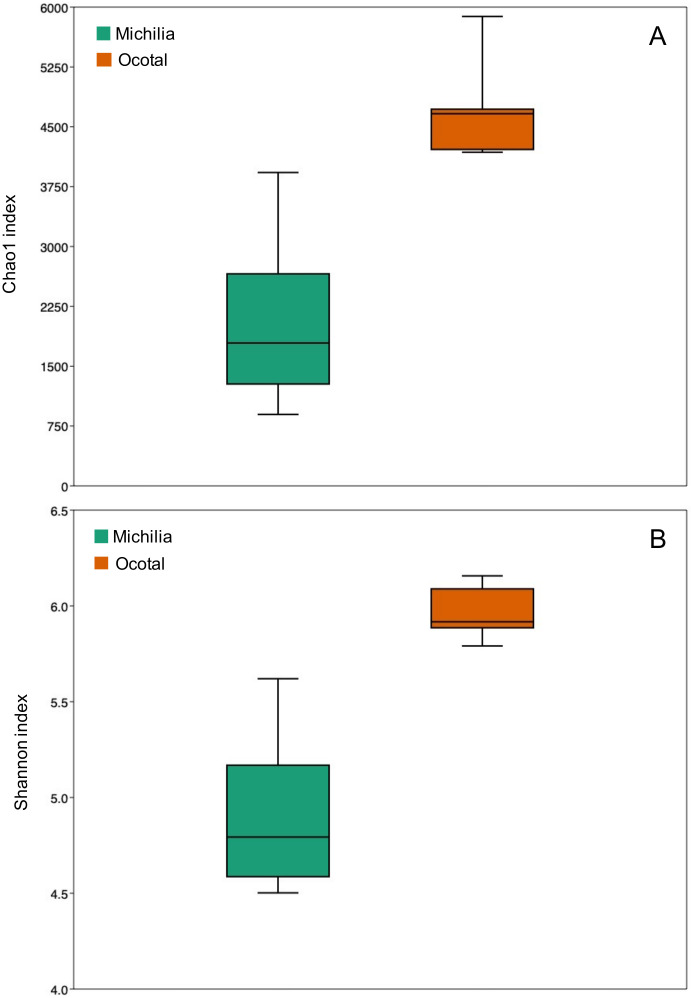
Boxplots of (**A**) Chao1 richness index and (**B**) Shannon alpha diversity index for the bacterial microbiota from two populations of Mexican wolves *(Canis lupus baileyi)* in Michilia (M, 23° N) and Ocotal (O, 19° N), Mexico.

**Figure 2:**
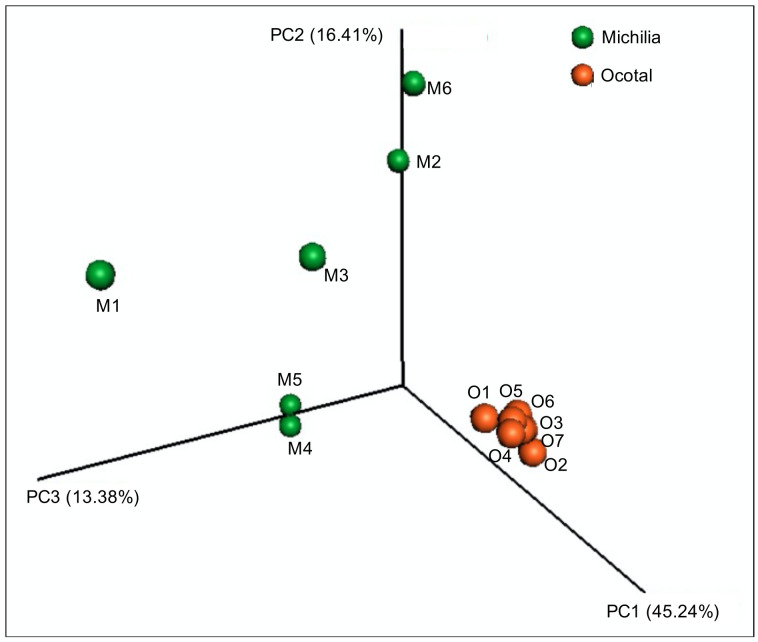
Principal Coordinate Analysis (PCoA) plot based on Bray–Curtis index of the fecal bacterial microbiota from two populations of Mexican wolves *(Canis lupus baileyi)* in Michilia (M, 23° N) and Ocotal (O, 19° N), Mexico.

The estimated Chao1 richness index differed (*p* < 0.001) between populations (t = 5.829, *p* < 0.001; Figure 1A). The mean Chao1 index for the Michilia samples was 2007.07 ± 1067.46 (SD), compared to 4705.56 ± 565.26 (SD) in Ocotal. Likewise, a significant difference was observed in the Shannon alpha diversity index (t = 6.538, *p* < 0.001; Figure 1B). While the mean Shannon index for the Michilia samples was 4.89 ± 0.41 (SD), a greater index occurred in the Ocotal (5.95 ± 0.12 (SD)). Differences also occurred between populations regarding the Bray–Curtis beta diversity matrix (PERMANOVA: pseudo-F = 7.63; *p* = 0.002; Figure 2). These results indicated that the group segregation is considerable, confirming a significant difference in the bacterial community structure between populations.

**Figure 3:**
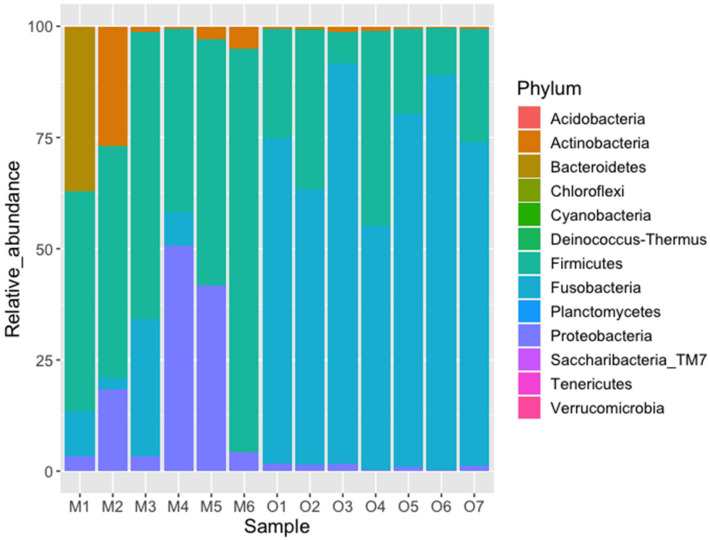
Relative abundance (%) of fecal bacterial taxa (phylum level) from two populations of Mexican wolves (*Canis lupus baileyi*) in Michilia (M, 23° N) and Ocotal (O, 19° N), Mexico.

**Figure 4:**
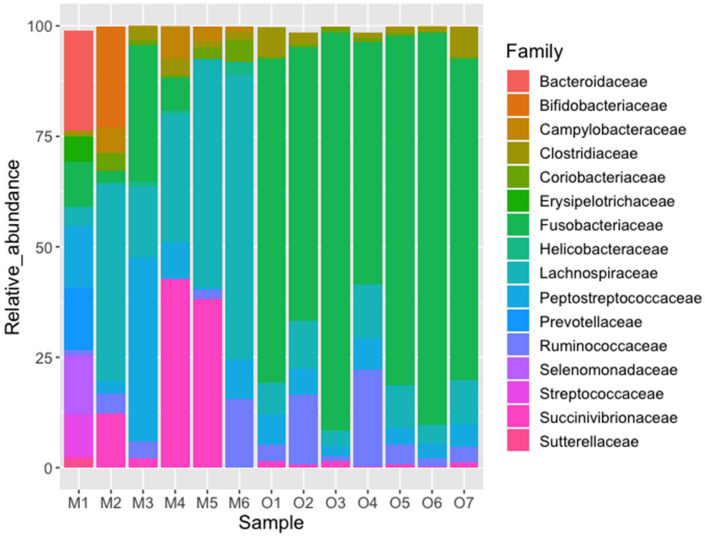
Relative abundance (%) of the fecal bacterial taxa (family level) from two populations of Mexican wolves (*Canis lupus baileyi*) in Michilia (M, 23° N) and Ocotal (O, 19° N), Mexico; only the first 16 more abundant families are shown.

The most abundant phyla were Firmicutes ($\bar{x}$ = 58.9%) and Proteobacteria ($\bar{x}$ = 20.2%) in the Michilia population, and Fusobacteria ($\bar{x}$ = 74.4%) and Firmicutes ($\bar{x}$ = 23.7%) in the Ocotal population (Figure 3). The SIMPER analysis showed a global dissimilarity between both groups of 66.03, with three phyla contributing with 91.07% of the difference (Fusobacteria, Firmicutes, and Proteobacteria), Fusobacteria being more abundant in Ocotal, while Firmicutes and Proteobacteria in Michilia (Table 3). At the family level, the most abundant taxa in Michilia were Lachnospiraceae ($\bar{x}$ = 34.9%), Succinivibrionaceae ($\bar{x}$ = 15.9%), and Peptostreptococcaceae ($\bar{x}$ = 12.6%). Regarding the Ocotal, the most abundant were Fusobacteriaceae ($\bar{x}$ = 74.4%), Lachnospiraceae ($\bar{x}$ = 8.1%), and Ruminococcaceae ($\bar{x}$ = 7.5%) (Figure 4).

**Figure 5:**
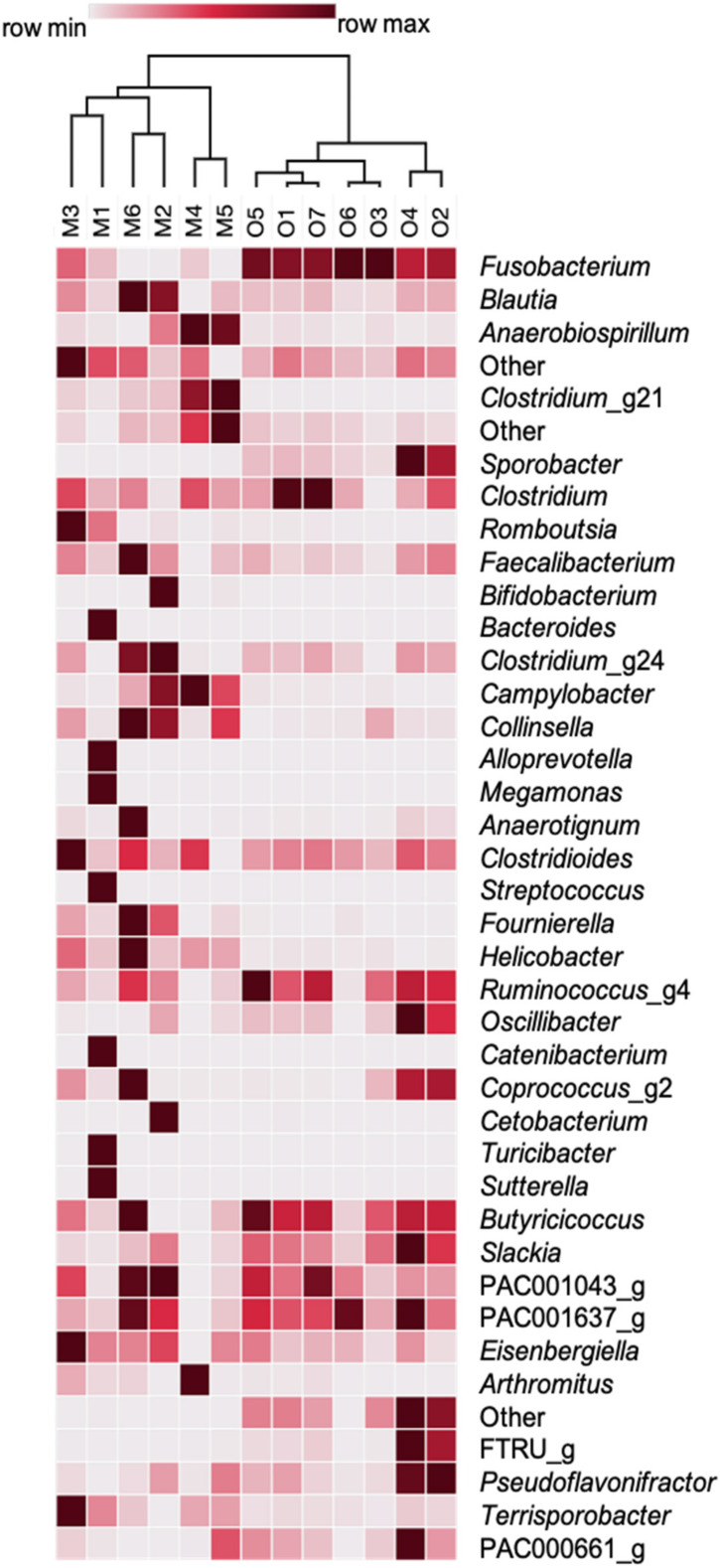
Heatmap of the fecal bacterial taxa (genera level) from two populations of Mexican wolves (*Canis lupus baileyi*) in Michilia (M, 23° N) and Ocotal (O, 19° N), Mexico; only the first 40 more abundant genera are shown.

The SIMPER analysis showed an overall dissimilarity of 74.83 between populations. Fusobacteriaceae were more abundant (*p* < 0.05) in Ocotal, while Lachnospiraceae, Campylobacteraceae, and Helicobacteraceae were more numerous in Michilia (Table 3). The most abundant bacterial genera in Michilia were *Anaerobiospirillum* ($\bar{x}$ = 15.9%), *Blautia* ($\bar{x}$ = 15.70%), and *Fusobacterium* ($\bar{x}$ = 8.1%), while in the Ocotal were *Fusobacterium* ($\bar{x}$ = 74.3%), *Sporobacter* ($\bar{x}$ = 4.6%), and *Blautia* ($\bar{x}$ = 4.5%) (Figure 5). The SIMPER analysis showed a global dissimilarity of 79.27 between populations, where the abundances of *Fusobacterium* and *Sporobacter* were higher (*p* < 0.05) in Ocotal, while *Clostridium*_g21, *Romboutsia*, *Campylobacter*, *Collinsella*, *Fournierella*, and *Helicobacter* were more abundant in Michilia (Table 3). The complete list of fecal bacterial microbiota (from phylum to species) for both Mexican wolf populations is shown in Table S1. The core microbiota analysis showed 11 bacterial genera for Michilia and 23 for Ocotal (Table 4).

**Figure 6:**
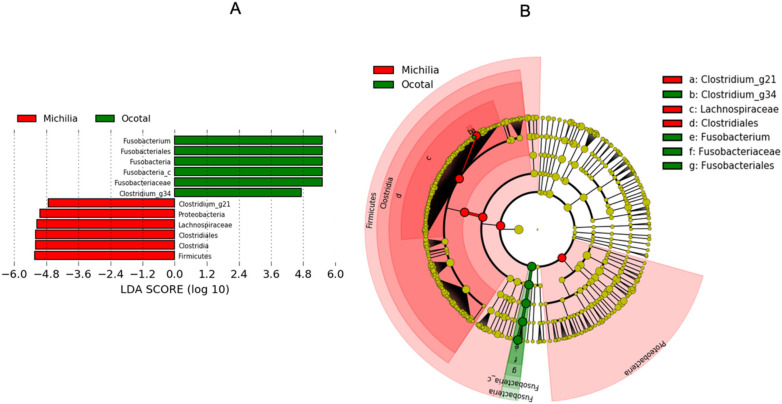
LEfSe analysis of fecal bacterial microbiota from two populations of Mexican wolves (*Canis lupus baileyi*) in Michilia and Ocotal, Mexico. (**A**) Bar graph shows LDA scores which indicate the taxonomic key for differentiation between populations. (**B**) The cladogram generated by LEfSe indicates the main biomarkers between populations. Each successive circle represents one phylogenetic level. Red-colored regions indicate taxa enriched in Michilia Mexican wolves, while green-colored regions indicate taxa enriched in Ocotal Mexican wolves.

LefSe analysis showed differences at taxa level between Michilia and Ocotal Mexican wolves. The bar graph from LefSe analysis (Figure 6A) displays LDA scores of microbial taxa with significant differences between populations. Firmicutes and Proteobacteria phyla, Clostridia class, Clostridiales order, Lachnospiraceae family, and *Clostridium*_g21 genus were enriched in the Mexican wolves of the Michilia population. For the Ocotal population, enrichment was observed in the Fusobacteria phylum, Fusobacteria_c class, Fusobacteriales order, Fusobacteriaceae family, and *Fusobacterium* and *Clostridium_*g34 genera. Principal biomarkers are shown in a cladogram (Figure 6B).

## 4. Discussion

It has been documented that abundance and diversity of the canine fecal microbiota is influenced by age, diet, health status, peer-social relationships, and anthropogenic-animal interactions, among other variables [9,34,35,36]. In the present study, the fecal bacterial microbiota of two Mexican wolf populations was evaluated. Although the geographical separation between populations is considerable, the type of habitat (temperate forest) in each locality was similar. Likewise, individuals from both populations have contact only with the personnel in charge of their care. However, Michilia and Ocotal differ in terms of grouping, age, and diet. While the Michilia wolves were separated either individually, or in pairs or trios (ages from 3 to 12 years old), Ocotal wolves lived together in a pack (average 3 yaers old). Regarding the diet, the wolves of Michilia were fed with kibble daily and raw meat sporadically, and in Ocotal the diet was based on raw meat daily and live animals periodically. Although it is probable that the type of interaction between individuals and the different ages may contribute to the difference in fecal microbiota observed in the present study, diet is considered a main factor closely related to the type of bacterial communities present in the intestine of vertebrates [10,12]; this variable will be discussed further.

Significant difference was observed in the fecal microbiota between populations, where the Ocotal wolves showed greater specific richness, higher within samples diversity of OTUs, and more than twice bacterial genera in its core microbiota compared to Michilia wolves. Previous studies have documented that wolves and dogs fed with raw meat show Bacteroidetes (46.4%), Fusobacteria (30.5%), Firmicutes (13.4%), Proteobacteria (8.8%) and Actinobacteria (0.5%) as the most abundant fecal bacteria at phylum level [8,9,10]. These same phyla were recorded as part of the fecal microbiota in the two studied Mexican wolves’ populations. However, in the Michilia wolves, an increased frequency of Firmicutes (58%) occurred, followed by Proteobacteria (20%); it must be highlighted that the diet offered to these animals was based on a low-quality kibble (low protein, 16%, and high carbohydrates, 57%), and eventually on raw meat. Therefore, the low-quality feed provided to these animals could explain the difference between the dominant phyla found and those expected if a high-quality meat-based or kibble diet had been provided. Diet studies on wolves and dogs have established that Firmicutes is abundant in canines eating diets based on low to medium-quality kibble or canned commercial food, due to its high carbohydrate content (30 to 60% starch), which favors proliferation of this phylum [11,12]. On the other hand, in the Ocotal wolves, the predominant phylum was Fusobacteria (i.e., 74%). In this population, the daily diet was raw meat, as well as live rabbits and guinea pigs weekly to encourage their hunt drives. Diets based on raw meat and BARF (i.e., Bones and Raw Food) are rich in proteins, increasing the abundance of Fusobacteria [8,34]. At the family level, the SIMPER analysis registered a greater abundance of Lachnospiraceae (phylum Firmicutes) in Michilia wolves, while a predominance of Fusobacteriaceae family (phylum Fusobacteria) was observed in the Ocotal wolves. Likewise, the LEFSE analysis showed that these two families are important biomarkers of the fecal microbiota in the analyzed populations. At the genus level, some of the taxa reported in previous canine studies were *Bacteroides, Blautia, Catenibacterium, Clostridium, Collinsella, Coprobacillus, Corynebacterium, Fusobacterium, Megamonas, Prevotella*, and *Turicibacter* [10,37,38,39]. These genera coincide with those registered as part of the Mexican wolf’s fecal microbiota in the present study. Nonetheless, the most abundant genera detected in Michilia wolves were *Anaerobiospirillum* and *Blautia*, accounting 15% of relative abundance. From these two genera, only *Blautia* was part of the core microbiota of this population, along with *Campylobacter, Clostrodium, Collinsella, Eisenbergiella, Helicobacter, Romboutsia*, and *Ruminococcus.* Interestingly, most of them belong to the phylum Firmicutes, indicating that, also at the genus level, the microbiota of these wolves has been conditioned to a diet rich in carbohydrates (57%). Conversely, in the Ocotal wolves, a dominance of the genus *Fusobacterium* was registered (74%), and the LefSe indicates that this taxon appears as a biomarker for this population. This genus has been profusely reported in canines fed raw meat [40] and has also been associated with healthy dogs living outdoors [41,42].

In general, wild canine species are adapted to a diet based on prey animals (high protein content) [43,44,45]. However, some conservation facilities have chosen to feed commercial dog products to captive wolves, due to their low cost and ease of handling compared to meat-based diets. In fact, commercial products can be an excellent option to preserve a good health status in these species, as long as these animals receive a high-quality product. On this respect, the Association of Zoos and Aquariums and the Animal Welfare Committee [7], recommend that commercial food for large canines (*Canis lupus* and its subspecies) should contain 20–28% protein, 5–10% fat, and 2–4% crude fiber. Specifically, for the Mexican wolf, the US Fish and Wildlife Service [46], recommends commercial food with 29% protein, 18% fat, 12% water, 9% ash, and 32% carbohydrates (Mazuri Exotic Canine Diet (5MN2)). When a low-quality kibble (like the one provided in Michilia) is offered, an alteration in the functional diversity of the intestinal microbiota could occur, generating gastric discomfort, loose/watery stools, or even diarrhea [8,47,48]. When evaluating the relationship between the consistency of feces and different types of diets in red wolf (*Canis rufus*), Bragg et al. (2020) [8], reported that the feces tended to be loose or even watery when the diet is based on low-quality kibble, and certain bacterial genera such as *Blautia* and *Romboutsia* were associated with this abnormal fecal condition. In our study, four (M1, M2, M3, and M6) of six fecal samples collected from the Michilia wolves were loose or watery (3.5 to 4 according to the Whaltham faeces scoring system [49]), while the genera associated to this fecal condition (i.e., *Blautia* and *Romboutsia*) were relatively abundant in these individuals. Additionally, according to Wu et al. (2017) [9], the proportion of Proteobacteria and Actinobacteria in wolves consuming appropriate diets is 8.8% and 0.5%, respectively. However, the Michilia wolves showed an average of 20% in Proteobacteria, while the M2-wolf showed 2.6% of Actinobacteria, indicating an imbalance of abundance in these phyla. Another study evaluated dogs of different ages, sexes, and breeds, and reported that changes in diet (from raw meat or cooked to kibble) led to an increase in the abundance of Proteobacteria [40,50,51]. Nonetheless, significant increases in these two phyla have been reported in dogs suffering from inflammatory bowel disease (IBD) [52,53]. It is possible that this imbalance in the Michilia wolves’ microbiota may have contributed to the observed watery stools, but the main concern is that this condition could lead to serious gastric disorders if the main problem (low-quality food) is not addressed. According to the facility staff, it is common to find loose feces from these wolves every day. Therefore, it is highly recommended that this facility considers changing to a higher quality food according to the nutritional guidelines provided by the US Fish and Wildlife Service [46]. All facilities that keep wild canines and other species of animals in captivity should follow management plans in which the health and welfare of all individuals is a priority.

## 5. Conclusions

The fecal bacterial microbiota of the Mexican wolf characterized in two populations, was affected by the different diets that the wolves have access to. In the Michilia wolves, with a diet based on high carbohydrate kibble and occasionally on raw meat, an increased frequency of Firmicutes and Proteobacteria occurred. On the contrary, in the Ocotal wolves, the most predominant phylum was Fusobacteria; these wolves were mainly feed with a high-protein diet, based on raw meat and live prey, increasing the abundance of Fusobacteria. It is recommended that the facilities where Mexican wolves are raised could maintain a constant evaluation of the diet provided to their animals, and improve it if necessary (e.g., high-quality kibble), to maintain the best possible digestive health in their individuals. While we still have a fragmentary knowledge regarding the main factors that shape the intestinal microbiota in the Mexican wolf, the main information generated in this study should help to enhance the welfare of these canines kept in captivity, while supporting diverse reproductive programs to increase its numbers. Being an endemic species of Mexico, not only the government authorities, but also the wildlife policy makers, civil organizations, and the scientific-academic community, have an enormous responsibility to reverse the damage that years ago placed this species on the brink of extinction. This astonishing while utmost endangered wolf species deserves our thoroughgoing commitment to do so, with the core aim to uphold a plentiful while ample recolonization of its former historical range; an undoubtedly pending assignment.

## Figures and Tables

**Table 1:** Fecal samples collected according to location, sex, age, and type of grouping from two populations of Mexican wolves *(Canis lupus baileyi)* in Michilia (M, 23° N) and Ocotal (O, 19° N), Mexico.

Location	Sex	Age (Years)	Grouping
M1	Female	3	Cohabitating with M2
M2	Female	3	Cohabitating with M1
M3	Male	12	Alone
M4	Male	2	Cohabitating with M5 and M6
M5	Male	2	Cohabitating with M4 AND M6
M6	Male	2	Cohabitating with M4 AND M5
O1	Male	3	Pack
O2	Male	3	Pack
O3	Male	1	Pack
O4	Female	5	Pack
O5	Female	5	Pack
O6	Female	1	Pack
O7	Female	1	Pack

**Table 2:** Fecal samples sequences obtained from two populations of Mexican wolves *(Canis lupus baileyi)* in Michilia (M, 23° N) and Ocotal (O, 19° N), Mexico.

Location	Total Reads	Assembled Reads	QB ^1^	OTUs ^2^
M1	142,533	42,013	35,441	735
M2	190,819	20,205	13,051	1074
M3	168,432	49,194	31,220	2537
M4	150,884	49,018	33,674	1836
M5	132,288	41,866	25,773	1631
M6	181,892	28,113	19,643	1038
Mean	161,141	38,402	26,467	1475
O1	119,488	46,215	28,913	3085
O2	141,153	61,695	43,526	3506
O3	140,534	64,251	44,318	3512
O4	151,679	76,461	54,132	3891
O5	119,588	51,454	34,252	3430
O6	128,670	57,787	35,222	3554
O7	89,352	38,777	18,936	3165
Mean	127,209	56,663	37,043	3449

^1^ QB = Quality bacterial sequences; ^2^ OTUs = operational taxonomic units.

**Table 3:** Percentage similarity analysis (SIMPER) considering the average of dissimilarity (AVD) of fecal bacteria at phyla, family, and genus levels from two populations of Mexican wolves (*Canis lupus baileyi*) in Michilia (M, 23° N) and Ocotal (O, 19° N), Mexico.

Taxon	AVD	Contribution %	Cumulative %	Mean M	Mean O	U	*p*-Value
Phylum							
Fusobacteria	32.93	49.87	49.87	0.085	0.744	0	0.001
Firmicutes	17.63	26.69	76.56	0.589	0.238	1	0.001
Proteobacteria	9.58	14.51	91.07	0.202	0.011	0	0.001
Bacteroidetes	3.07	4.64	95.72	0.061	0.000	14	0.353
Actinobacteria	2.81	4.26	99.98	0.062	0.007	13	0.283
Family							
Fusobacteriaceae	32.93	44.01	44.01	0.085	0.744	0	0.001
Lachnospiraceae	14.09	18.83	62.84	0.350	0.082	6	0.038
Succinivibrionaceae	7.75	10.36	73.20	0.160	0.009	12	0.224
Peptostreptococcaceae	4.92	6.57	79.77	0.126	0.049	13	0.283
Ruminococcaceae	3.30	4.41	84.18	0.046	0.075	15	0.432
Bifidobacteriaceae	1.94	2.59	86.77	0.039	0.000	-	-
Bacteroidaceae	1.89	2.52	89.29	0.038	0.000	14	0.350
Campylobacteraceae	1.43	1.90	91.19	0.029	0.001	5	0.026
Prevotellaceae	1.18	1.58	92.77	0.024	0.000	-	-
Clostridiaceae	1.14	1.52	94.29	0.019	0.027	20	0.943
Selenomonadaceae	1.13	1.51	95.80	0.023	0.000	-	-
Coriobacteriaceae	0.89	1.19	96.99	0.023	0.007	13	0.283
Streptococcaceae	0.77	1.03	98.02	0.015	0.000	-	-
Helicobacteraceae	0.46	0.62	98.64	0.010	0.000	0	0.003
Genus							
*Fusobacterium*	33.14	41.80	41.80	0.081	0.744	0	0.001
*Anaerobiospirillum*	7.75	9.78	51.58	0.160	0.008	12	0.224
*Blautia*	6.71	8.47	60.05	0.157	0.045	15	0.432
*Clostridium_*g21	4.50	5.68	65.73	0.091	0.001	0	0.001
*Other*	2.51	3.16	68.89	0.069	0.039	13	0.283
*Sporobacter*	2.31	2.91	71.80	0.000	0.046	0	0.001
*Romboutsia*	2.10	2.65	74.45	0.043	0.001	5	0.026
*Other*	2.06	2.59	77.04	0.048	0.011	13	0.283
*Bifidobacterium*	1.94	2.44	79.48	0.039	0.000	-	-
*Bacteroides*	1.89	2.38	81.86	0.038	0.000	14	0.350
*Campylobacter*	1.43	1.80	83.66	0.029	0.001	5	0.026
*Faecalibacterium*	1.20	1.52	85.17	0.029	0.013	16	0.520
*Alloprevotella*	1.18	1.49	86.66	0.024	0.000	-	-
*Megamonas*	1.13	1.43	88.09	0.023	0.000	-	-
*Clostridium*	1.08	1.37	89.45	0.016	0.027	20	0.943
*Anaerotignum*	1.01	1.28	90.73	0.020	0.002	19	0.829
*Collinsella*	0.90	1.14	91.87	0.022	0.005	6	0.038
*Clostridium_*g24	0.84	1.06	92.93	0.021	0.010	19	0.830
*Streptococcus*	0.76	0.96	93.90	0.015	0.000	-	-
*Fournierella*	0.62	0.78	94.68	0.013	0.000	6.5	0.045
*Helicobacter*	0.46	0.58	95.26	0.010	0.000	0	0.001
*Clostridioides*	0.45	0.56	95.83	0.012	0.008	20	0.943
*Oscillibacter*	0.20	0.26	96.35	0.001	0.004	9	0.100
*Turicibacter*	0.17	0.22	96.81	0.003	0.000	-	-

**Table 4:** Core fecal bacterial microbiota (taxa that were found in all individuals) from two populations of Mexican wolves (*Canis lupus baileyi*) in Michilia (M, 23° N) and Ocotal (O, 19° N), Mexico.

	Core Microbiota	
Genera/Species	Michilia	Ocotal
*Agathobaculum*	*-*	*
*Anaerobiospirillum*	*-*	*
*Blautia*	*	*
*Butyricicoccus*	*-*	*
*Campylobacter*	*	*
*Clostridium*	*	*
*Clostridium_*g21	*	*
*Clostridium_*g24	*	*
*Collinsella*	*	*
*Eisenbergiella*	*	*
*Faecalibacterium*	*-*	*
*Faecalimonas*	*-*	*
*Fusobacterium*	*-*	*
*Fusobacterium necrophorum*	*-*	*
GU302778_g	-	*
*Helicobacter*	*	*
PAC001043_g	*	*
PAC001200_g	-	*
PAC001637_g	-	*
*Romboutsia*	*	-
*Roseburia*	*-*	*
*Ruminococcus_*g4	*	*
*Slackia*	*-*	*
*Sporobacter*	*-*	*

(*) denotes presence, (-) denotes absence.

## Data Availability

Fecal bacterial sequences are available on NCBI Bioproject PRJNA721093; SRA (SRP315294).

## Supplementary Materials

The following are available online at https://www.mdpi.com/article/10.3390/biology10070637/s1, Table S1: Relative abundance of fecal bacterial microbiota for two populations of Mexican wolves (*Canis lupus baileyi*) in Michilia (M, 23° N) and Ocotal (O, 19° N), Mexico. This table shows all fecal bacterial taxa (from phylum to species) obtained for both Mexican wolves (*Canis lupus baileyi*) populations (Michilia and Ocotal).
